# Job Stress across Gender: The Importance of Emotional and Intellectual Demands and Social Support in Women

**DOI:** 10.3390/ijerph10010375

**Published:** 2013-01-14

**Authors:** Pilar Rivera-Torres, Rafael Angel Araque-Padilla, María José Montero-Simó

**Affiliations:** 1 University of Zaragoza, C/ Gran Vía, 2, Zaragoza 50005, Spain; E-Mail: privera@unizar.es; 2 ETEA-University of Córdoba, C/Escritor Castilla Aguayo, 4, Córdoba 14004, Spain; E-Mail: raraque@etea.com

**Keywords:** job demands-control-support model, job strain, gender, Structural Equation Modelling (SEM), multi-sample analysis

## Abstract

This study aims to analyse whether any differences exist between the genders with respect to the effect of perceived Job Demands, Control and Support (JDCS model) on how individuals reach high levels of job stress. To do this, the perceived risk of suffering an illness or having an accident in the workplace is used as an outcome measure. The study is based on the First Survey on Working Conditions in Andalusia, which has a sample of 5,496 men and 2,779 women. We carry out a multi-sample analysis with structural equation models, controlling for age and sector. The results show that the generation of job stress has a different pattern in men and women. In the case of men, the results show that only one dimension of the job demands stressor is significant (quantitative demands), whose effect on job stress is weakened slightly by the direct effects of control and support. With women, in contrast, emotional and intellectual aspects (qualitative demands) are also statistically significant. Moreover, social support has a greater weakening effect on the levels of job stress in women than in men. These results suggest that applying the JDCS model in function of the gender will contribute to a greater understanding of how to reduce the levels of job stress in men and women, helping the design of more effective policies in this area.

## 1. Introduction

Excess stress is the cause of considerable problems in developed countries. Much of this stress has been linked to work and employment [1], and the latter takes up a large part of our lives. According to the European Agency for Safety and Health at Work [2], stress was the second most common work-related health problem in 2005, when it affected 22% of workers in the EU-27. The Agency predicts that the number of people suffering stress-related conditions caused or made worse by work is likely to increase. Factors such as downsizing and outsourcing, the increasing need for flexibility in functions and skills, the increasing number of temporary contracts, the growing job insecurity, and poor work-life balance are imposing increasingly severe demands on workers, which is leading to greater tensions.

Occupational stress can transcend the workplace and also endanger the general wellbeing of the worker. A large number of studies link occupational stress to other health problems such as musculoskeletal disorders [3,4,5], cardiovascular disease [6,7], anxiety and depression [8,9,10], burnout [11,12,13,14], and insomnia [15,16]. Stress also has an important effect on the firm’s performance, in particular on creativity [17], productivity [18,19], innovation [20,21], commitment [22], and leadership [23,24].

Many of the factors that generate stress—or stressors—are psycho-social in nature. Moreover, it is generally accepted in the literature that people react differently to exposure to these factors. In other words, stress-related symptoms or illnesses can vary between individuals. Thus it is also important to consider gender when studying stress-related problems.

Women and men are exposed to different working environments and different types of demands and tensions, even when they work in the same sector and profession. Men are more likely to occupy higher positions. Women (42% of the active population in the EU) are more likely to work part-time than are men (32% of women say they work part-time, compared to 7% of men), and many women work in low-paid, precarious jobs, which affects their working conditions and hence the risks to which they are exposed. Women also tend to remain in the same job longer than men, so their exposure to any existing risks is longer-lasting. Consulting workers and their participation is an important factor in occupational health and safety, but women tend to work in jobs where union representation is lower. Finally, women still do the lion’s share of the unpaid work in the home and caring for children and relatives, even when they work full time. This is on top of their daily paid work and generates even more pressure, particularly when they cannot possibly reconcile work and family life [25].

A number of studies focusing on either stressors or their manifestations analyse the influence of gender on the levels of job strain in the workplace, but the situation in the workplace as discussed above seems to make it necessary to deepen the analysis.

In the current study we apply the JDCS model on a large sample of workers to investigate whether men and women differ significantly in their perceptions of Demands, Control and Support in the workplace and in how these factors affect the levels of job stress. To do this, the perceived risk of suffering an illness or having an accident in the workplace is used as a manifestation of job stress.We also examine whether the socio-demographic variables age and sector of activity affect job stress differently in function of the gender.

## 2. Theoretical Framework

### 2.1. Gender and Occupational Stress

Research shows that occupational stress can affect both men and women. Nevertheless, women may be disproportionately exposed to stressors. Women have greater exposure to monotonous tasks than men, are less likely to do jobs involving problem solving or learning, are less likely to be able to choose when to take a break in their work, and are more likely to be interrupted with unexpected tasks [26,27,28].

The literature review for this study suggests that the genders do not differ for all manifestations of occupational stress. For example, researchers find no differences between women and men in terms of the influence of stress factors on perceived role conflicts [29], personal accomplishment [30], or self-esteem or well-being [31]. The relation between burnout and gender aspects in general is unclear [32]: some studies find that women suffer burnout more than men, but others come to the opposite conclusion. This lack of consensus may be because researchers have shown that burnout is closely linked to the profession, and this is what determines its intensity.

Research has found significant differences between men and women with regards harmful job strain or its effects on other symptomatic variables. Women seem to suffer more from problems such as mental disorders, depression, anxiety and psycho-somatic illnesses [31,33,34,35], while men suffer more from heart disease, which is caused by a number of occupational factors, including stress [36]. Other research [20,21] suggests that stress has a stronger negative impact on aspects such as innovative behaviour in the workplace among women, and a weaker negative effect on others such as personal realisation [37], exhaustion or depersonalisation [30]. Researchers have also found a gender effect in the perception of stress in general. Thus men seem to experience higher levels of stress [9,34,38].

### 2.2. The Job Demands-Control-Support (JDCS) Model

Some stressors are psycho-social factors. An extensive literature—in medicine as well as psychology and sociology—links these stressors with health problems among workers. One of the dominant models in the area of psycho-social occupational stress is the Job Demands-Control (JDC) model [39,40]. This model predicts that the highest job stress is experienced in environments characterised by high job demands and low job control. Later, this model was expanded in the Job Demands-Control-Support (JDCS) model [6]. This model’s central hypothesis is that job stress increases as job demands increase and as control and support levels decrease. Thus stress will conceivably have more damaging effects on health in jobs with high demands, and low control and social support. In contrast, stress will conceivably have a less harmful impact on health in jobs with fewer demands and a high level of control and social support. The literature has yet to reach a consensus about the buffer effects of control and support, and the interactions between different types of demands (quantitative and qualitative), control and support.

A large number of studies validate the capacity of the JDCS model to explain the adverse effects of job stress on health [29,37,41,42,43,44,45,46,47]. Many of these introduce gender as a control variable, and their results differ on various levels. We have noted above some of the differences between women and men with regards the effects of stress.

The above differences can be explained, within the theoretical framework of the JDCS model, by the fact that men and women differ in their perceptions about the influence of the various psycho-social factors on mental stress. Though here too we find a number of discrepancies between the different studies reviewed. Some authors find that gender has an important influence on the three dimensions of the JDCS model [34]. But others find differences in only some of the dimensions. Grönlund [48] concludes that job demands and control have similar effects for both genders, contrary to other authors [49,50] who point out that characteristics of the workplace, such as job demands and job control, may have a greater impact on psychological well-being among men. Other studies find differences between the genders only with regards the perceived level of control in the workplace [20,51,52,53], but not with regards job demands, unlike other research [9,21,37], or support. But Sanne *et al.* [9] find that support has a stronger buffer effect among women.

Other studies link the effect of the psycho-social factors to occupational status. Burke [35] finds that stressors have the strongest influence in women working in lower-status jobs. This result is consistent with Grönlund’s [48] findings about psycho-social factors and work-family conflict. This author finds that women are more likely to have this type of stressful work than men. But she also concludes that no differences exist between men and women in jobs with high demands and a high level of control.

The studies reviewed for this work offer inconclusive results with regards the impact of psycho-social factors in women and men, as well as whether the two groups have different perceptions about these factors. Thus the studies have not reached a consensus about whether women and men differ with regards the problems caused by stress and their perception of the stressors. We should mention another aspect that has been less researched in the literature on gender and occupational stress: the relative potential of the different stressors to generate stress, for men and women. Is stress generated in the same way in both genders? Do job demands, control or support have the same capacity to generate job strain in both genders?

In this work we try to respond to these questions using the perceived risk of suffering an illness or having an accident as a manifestation of job strain. We are particularly interested in looking for significant differences between the genders in the relative impact of the JDCS model factors on the levels of job strain on job strain. If empirical support were found for these differences, the design of occupational risk management policies could be improved by adapting them in function of gender.

## 3. Methodology

### 3.1. Sample: Survey on Working Conditions in Andalusia

The current research takes as starting point a study on working conditions in Andalusia, an autonomous region in southern Spain, in 2008. That study involved a general questionnaire on health and safety in the workplace, and one of the aspects analysed was psycho-social risks. The population under analysis consisted of all workers living in towns with a population of over 5,000 people in Andalusia. The sample was formed using multi-stage stratified random sampling. It was first stratified by province and sector of activity, and then by gender and firm size. The data collection was via personal interviews in the respondent’s home (using the random-route method), and the final sample consisted of 8,275 respondents from 139 towns. Given the subsequent use of multivariate analysis we used listwise deletion to deal with the missing data. Thus the data matrix contained 7,512 elements.

### 3.2. Measurement Scales

The measurement instrument on which this research is based is the First Survey on Working Conditions in Andalusia carried out by the Andalusian Institute for Occupational Health and Safety, part of the Employment Ministry of the Regional Government of Andalusia.

Thus this was not a tool specifically designed to measure groups of psycho-social factors (e.g., FPSICO or ISTAS21 in Spain), but a bigger tool designed to measure other work-related characteristics.

After analysing the different proposals in the literature for measuring psycho-social stressors, and on the basis of the abovementioned survey on working conditions in Andalusia, we selected 10 indicators for the concepts of Quantitative demands, Qualitative demands, Control and Support, and two indicators for the concept of Perceived risk as a manifestation of Job strain.

For the Demands construct we chose two indicators from the questionnaire that measure Quantitative demands (work overload)—Work fast and Time pressure [5,12,20,39,44,45]—and two indicators measuring Qualitative demands—Intellectually demanding job and Emotionally demanding job [12,21,22,41,42]. For Control, we chose four indicators representing the Authority dimension: Freedom to decide the order in which you do things; Freedom to choose your own method of working; Freedom to decide the pace of work; and Freedom to choose or change the distribution/duration of pauses [12,21,29,34,39,41,42,43,44,45,46,48]. We used two items to measure Support: Co-worker support; and Supervisor support [12,21,22,43,44,45].

To operationalise the dependent variable (expression of Job stress) we used two indicators: Perceived risk of having an accident in the workplace; and Perceived risk of suffering an illness due to work. These indicators were measured in the questionnaire using a 5-point Likert scale with the following question: 1) To what extent does the following aspect of work worry you: the risk of having an accident, and 2) To what extent does the following aspect of work worry you: the risk of suffering an illness. In this study, the choice of subjective rather than objective indicators for evaluating job stress was conditioned by the source of the information that is analysed, namely a self-administered questionnaire. We chose perceived risk in the workplace as a manifestation of Job stress rather than other possible health problems because of the importance of this factor in predicting behaviours relating to preventive behaviour. In the labour sphere it is postulated that perceived risk conditions the worker’s preventive reaction or conduct [54,55]. Although it is necessary for an objective labour risk to exist for an accident to happen, we should not underestimate subjective risk, because workers often detect the existence of real risks that cannot be determined objectively or scientifically. In short, if the worker subjectively perceives the existence of a risk, although objectively it does not exist, the worker will behave as if the risk really exists [56].

However, accepting the importance of analysing perceived risk in the workplace, it was necessary to examine whether the individuals who experience a greater Job stress also tend to perceive a greater risk of suffering an illness or having an accident as a manifestation of this. The authors did this in a previous work [57], using the JDCS model. The results of that work provide support for the validity of using the two Perceived risk indicators as a manifestation of Job strain.

All the indicators of the concepts Demands, Control, Support and Perceived risk were measured on 5-point Likert scales. In all the indicators, a higher score means a higher level of the attribute or characteristic in question.

The variables age and sector were defined as control variables because of their widely supported importance in explaining differences in both the dependent and independent variables [13,20,29,34,37,41,42,44,45,46,48,58,59]. The variable Age is the age of the subject, in years. The variable sector is a categorical variable, having four levels (industry, construction, services and agriculture). Thus we created three dummy variables (construction, services and agriculture) and the base sector was industry.

### 3.3. Procedure

In order to analyse, from the gender perspective, “the psycho-social stressors of the JDCS model and their effects on subjects’ perceived risk of suffering an illness or having an accident in the workplace”, we first carried out a univariate analysis of the indicators for both samples. We analysed with the z-test whether the mean perceptions differ statistically between the two samples. The sample consists of 7,512 complete records, which reduces Type II error, but the ability to detect a small difference increases. Consequently, in this research we accepted a maximum Type I error of 1%.

The next step was to analyse the correlation matrix of the 12 indicators. This matrix was used to start to evaluate the dimensionality structure of the psycho-social stressors of the JDCS model (Quantitative demands, Qualitative demands, Control, Support) and the Perceived Risk for both samples. Following this analysis, we tested this structure with a Confirmatory Factor Analysis using Structural Equation Modelling (SEM) by a multi-sample perspective. This is because the effects of the psycho-social stressors of the JDCS model on subjects’ perceived risk in the workplace cannot be tested unless this factor structure is confirmed across the two samples.

Once the measurement models—the factor structures of the relations between the indicators and their psycho-social stressors and the perceived risk—were validated in both samples, the Structural Model was analysed using SEM. The Structural Model presents the four effects of the psycho-social stressors of the JDCS model on subjects’ perceived risk. In this model we incorporated two socio-demographic characteristics as control variables: age and sector.

After the eight structural parameters were estimated independently in both samples—the four effects of the psycho-social stressors of the JDCS model and the four effects of the control variables on subjects’ perceived risk—we carried out the eight pertinent tests to determine differences in the structural parameters for independent samples. This made it possible to evaluate whether “the effects of the psycho-social stressors of the JDCS model and the socio-demographic variables on subjects’ perceived risk in the workplace” differ statistically between the two samples. We accepted a maximum Type I error of 1%.

The software application used to estimate the Confirmatory Factor Analysis and the Structural Equation by a multi-sample perspective was EQS 6.2 [60]. In this research the assumption of normality cannot hold for the data, so we used the Maximum Likelihood (ML) estimation method with the “robust” covariance matrix and the correct z-tests [61]. For the purpose of evaluating the measurement and structural models we analysed the Satorra-Bentler Robust χ^2^ [61]. This statistic is affected by the sample and model size. Consequently, the following are considered at the same time: the Robust Root Mean Square Error of Approximation (R-RMSEA); the Standardised Root Mean Square Residual (SRMR); the Goodness-of-Fit Index (GFI); and the Adjusted Goodness-of-Fit Index (AGFI) [62]. In order to evaluate the construct validity of the four psycho-social stressors of the JDCS model and the subjects’ perceived risk we used the standardised factor loadings. The standardised factor loadings are statistically significant and sufficiently large when λ > 0.70 [63]. Two coefficients used to measure the precision for each of the constructs were: the composite reliability coefficient (CRC) [64], and the average variance extracted (AVE) [65]. The recommended values for CRC and AVE are above 0.70 and 0.50, respectively [63].

A multi-sample analysis involves analysing a model in several samples simultaneously. This not only allows researchers to test whether the model fits the data for any single sample, but also to test differences between the samples in the model parameters. The Lagrange Multiplier Test (LMTEST) was used to evaluate the equality of the parameters of the structural models across the two samples [66]. We accepted a maximum Type I error of 1%.

## 4. Results

Table 1 reports the means of the indicators in the group of women and in the group of men. We measured the variables using 5-point Likert scales: high (low) scores indicate high (low) levels of the attribute. The mean scores exceed 2.0 in all cases, and only the support indicators always exceed 3.5. In order to discover gender differences in the perception of psycho-social factors, we carried out difference of means z-tests for independent samples (Table 1). Looking at the results we can conclude that men perceive greater pressure from quantitative demands in the workplace (Work fast: 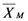 = 3.54 > 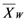 = 3.45; Time pressure: 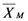 = 3.23 > 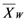 = 3.05), while women perceive greater pressure from qualitative or psychological demands (Intellectually demanding job: 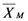 = 3.10 < 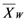 = 3.31; Emotionally demanding job: 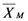 = 3.04 < 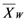 = 3.27). For control and support, no significant differences exist. Finally, men are generally more pessimistic than women with regards the risk of suffering an illness (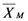 = 2.64 > 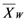 = 2.43) or having an accident (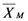 = 2.91 > 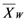 = 2.44) in the workplace.

The next step was to analyse the correlation matrix (12 indicators). The correlations among the indicators of the five dimensions in the two samples and the Cronbach alphas (in all cases these are higher than 0.70) provide evidence for the existence of five latent variables (Quantitative demands, Qualitative demands, Control, Support and Perceived risk). Thus in order to evaluate the dimensional structure in confirmatory terms, a first-order Confirmatory Factor Analysis was estimated with five latent variables for each sample. The R-RMSEA values are below 0.05, the SRMR values below 0.03, and the GFI/AGFI above 0.95, providing evidence to not reject the dimensional structure proposed. With regards the evidence for the reliability and validity of the dimensions, the factor loadings of the indicators for each construct are statistically significant and sufficiently large. Moreover, the coefficients also have a clear relation with the underlying factor (R^2^ > 0.50). For all latent variables, the CRC is above the recommended 0.70 and the AVE exceeds 0.50. These results, which are consistent with the correlation analysis that was performed, confirm the factor structure in both samples: Quantitative demands, Qualitative demands, Control, Support and Perceived risk.

**Table 1:** Descriptive statistics: mean, by gender.

**QUANTITATIVE DEMANDS (QND)**		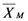	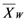	
QND1	Work fast	3.54	3.45	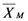 > 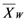 *
QND2	Time pressure	3.23	3.05	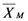 > 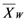 *
**QUALITATIVE DEMANDS (QLD)**				
QLD1	Intellectually demanding job	3.10	3.31	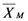 < 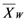 *
QLD2	Emotionally demanding job	3.04	3.27	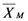 < 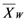 *
**CONTROL (CON)**				
CON1	Freedom to decide the order in which you do things	3.03		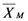 = 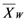
CON2	Freedom to choose your own method of working	2.93		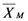 = 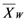
CON3	Freedom to decide the pace of work	2.90		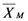 = 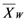
CON4	Freedom to choose or change the distribution/duration of pauses	2.89		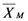 = 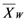
**SUPPORT (SUP)**				
SUP1	Co-worker support	4.20		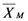 = 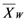
SUP2	Supervisor support	3.73		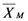 = 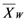
**PERCEIVED RISK (PR)**				
PR1	Perceived risk of having an accident in the workplace	2.91	2.44	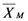 > 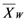 *
PR2	Perceived risk of suffering an illness due to your work	2.64	2.43	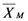 > 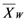 *

* p-value < 0.01.

**Figure 1:**
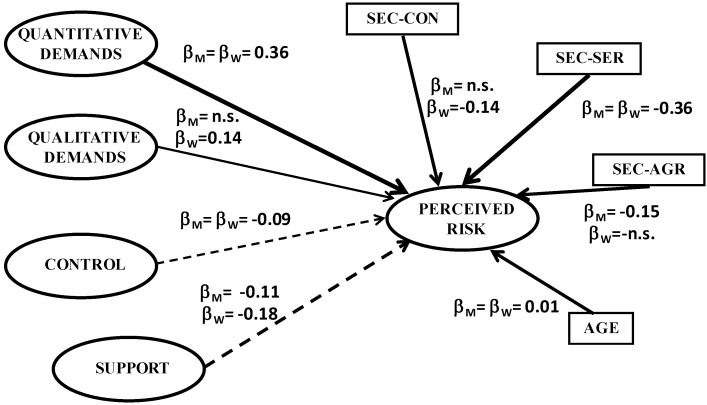
Structural Model.

After obtaining the results from the Confirmatory Factor Analysis, the next step was to analyse the structural relations between the psycho-social stressors of the JDCS model and the socio-demographic variables (age and sector) and the subjects’ perceived risk in the workplace across the two samples. The goodness-of-fit statistics (the R-RMSEA values are below 0.05, the SRMR values below 0.03, and the GFI/AGFI above 0.95) mean that we cannot reject the structural model under the assumption of inequality of parameters in the two samples (with free structural parameters across the two samples). Thus, in order to evaluate whether the differences observed in the structural parameters are significant or not, we ran the LMTEST. After imposing the equality constraints in the eight regression coefficients and looking at Figure 1 we can conclude that four of the eight constraints can be rejected. Therefore, we can conclude that the genders do not differ significantly with regards the effects on the subject’s perceived risk of quantitative demands (β = 0.36), perceived control (β = −0.09), age (β = 0.01) and services sector (β = −0.36). In contrast, men and women do differ significantly with regards the effects of qualitative demands (β_M_ = n.s. and β_W_ = 0.14), perceived support (β_M_ = −0.11 and β_W_ = −0.18) and the construction sector (β_M_ = n.s. and β_W_ = −0.14) and agriculture sector (β_M_ = −0.15 and β_W_ = n.s.) in comparison to industry.

## 5. Discussion and Conclusions

The aim of this work has been to study, by means of the JDCS model, to what extent significant differences exist between the genders in how individuals reach high levels of job stress. More specifically, we asked ourselves whether the dimensions of the model were equally significant and exert an influence with the same weight upon job stress. The results of the analysis indicate that significant differences do exist between the genders. 

With regards the dimensionality of the model, after the multi-sample analysis, we can conclude that the JDCS model is valid for explaining the perceived risk of suffering an illness or having an accident in the workplace in both women and men. In both cases the three dimensions postulated theoretically—Demands, Control and Support—are significant, with positive loads in the first case and negative ones in the following two. Nevertheless, we should mention that for women, two independent dimensions of Demands are significant—Quantitative and Qualitative demands—while for men, the Demands dimension refers only to quantitative, not qualitative, stressors. Thus both quantitative and qualitative (intellectual and emotional) demands determine occupational stress in women, while only quantitative demands are stressors for men. By differentiating between quantitative and qualitative demands we have been able to obtain a difference between the genders that has not been found in previous studies.

With regards the Quantitative demands, the results of the models show that this type of demand has similar effects on job stress among women and men. This finding contradicts Vermeulen and Mustard and Van der Doeff [49,50], who find that Quantitative demands carry a greater weight in the case of men.

Looking at more specific aspects and in particular at the analysis of means carried out, men have the perception of greater pressure from quantitative demands than women, while women feel a greater pressure from qualitative or psychological demands. These differences can be extrapolated to both populations. The greater perception of quantitative demands found in the case of men supports the work of Sanne *et al.* [9], but contradicts the findings of Landbergis [37].

Control reduces the level of stress in the workplace. As in the case of Quantitative demands, no significant differences exist between men and women with regards this effect. This result is consistent with Grönlund [48], although we must point out that in other studies Control usually has a greater weight in the case of men [49,50,52,53]. The fact that our work finds no differences may be due to the heterogeneity of occupations, and hence of responsibilities, among the respondents.

According to our results, women and men do not differ in their perceptions of the level of Control, in contrast to the findings of other studies [9,20,51].

The model presented here postulates that the Support dimension reduces job stress. Looking at the results here, a difference exists between men and women in the sample analysed. Support has a significantly stronger impact on levels of job stress in the workplace among women. This result is consistent with the findings of Sanne *et al.* [9]. At the same time, no differences exists between the groups with regards the level of perceived support.

These results taken as a whole do provide support for the claim that it is advisable to apply the JDCS model in samples that are differentiated by gender. More particularly, they highlight the importance of considering aspects such as Qualitative demands and Social support if we wish to understand how women reach high levels of job stress.

Our study offers several practical implications for designing organisational policies aimed at reducing harmful job stress among workers. Because the quantitative demands are the main stressor in both men and women, managing this type of demand should be the cornerstone of any preventive policy in both groups, whether via an improved organisation of tasks by the management, or encouraging improvements in time management among the workers. For women, stress prevention would also be improved by a better management of qualitative demands and by social support. In the first case, we should note that this study shows that women on average tend to perceive greater intellectual and emotional demands than men in the workplace. With regards the intellectual demands, if women perceive that they have to make greater efforts to prove their worth than men, this could be the cause of greater stress. It would then be necessary to investigate the extent to which this is due to discriminatory behaviours in the firm or perceptions based on preconceived ideas about the role of women in the workplace. With regards the greater perception of emotional demands in women, and their greater effect on stress, both findings may be related to women’s generally greater emotional involvement in social relationships. Although this involvement undoubtedly has positive social effects, the stress generated could be ameliorated via stronger social support from workmates and supervisors. We recall that support has a significantly stronger effect on stress in women than in men. This support could be encouraged by a more suitable design of the workgroups. It would be interesting to analyse whether men and women perceive greater support at work from people of the same or opposite sex.

Our work is, inevitably, not free of limitations. First, attention should be drawn to the measuring instrument. Not applying an *ad-hoc* questionnaire has prevented us from using a greater number of items to measure the different dimensions of the model. Nevertheless, we have attempted to offset this limitation by means of the methodological approach used and with the evidence from the literature regarding the content validity of the items selected. The methodology used was Multi-Group Structural Equation Models, and so the measurement error in the indicators and the checks for parameter equality were taken into account in both of the groups under consideration. Second, given its subjective nature, the use of the perceived risk of suffering an illness or accident in the workplace as an indicator of mental strain could distort the results. Nevertheless, the use of subjective measures can provide knowledge that is closer to the psychology of the individual. Third, in this study only the direct effects of Control and Social support have been taken into account, without considering possible moderating effects. Future work should address both types of effect in modelling job stress. 

This study suggests some other possible lines of research. On the one hand, it would be a good idea to conduct in-depth studies that differentiate not only between the genders but also between sectors and job categories. On the other hand, further research should be carried out on whether the differences between men and women undergo variations depending on the variable that is used as an indicator or expression of mental stress in the job.

To end, the aim of this work has been to examine in greater depth the role played by gender in the JDCS model and how it affects the perceptions of risk from the perspective of multi-group analysis. Accordingly, this research not only considers the effect of gender on the mean levels of Demands, Control and Support and Perceived risk in the workplace, but also examines the effect of gender on the relations between the psycho-social factors and the levels of job stress.
